# Preoperative Gadoxetic Acid-Enhanced MRI Based Nomogram Improves Prediction of Early HCC Recurrence After Ablation Therapy

**DOI:** 10.3389/fonc.2021.649682

**Published:** 2021-05-21

**Authors:** Chengguang Hu, Yangda Song, Jing Zhang, Lin Dai, Cuirong Tang, Meng Li, Weijia Liao, Yuchen Zhou, Yikai Xu, Yong-Yuan Zhang, Yuanping Zhou

**Affiliations:** ^1^ Guangdong Provincial Key Laboratory of Gastroenterology, Department of Gastroenterology, Nanfang Hospital, Southern Medical University, Guangzhou, China; ^2^ Department of Infectious Diseases and Hepatology Unit, Nanfang Hospital, Southern Medical University, Guangzhou, China; ^3^ Department of Radiology, Nanfang Hospital, Southern Medical University, Guangzhou, China; ^4^ Laboratory of Hepatobiliary and Pancreatic Surgery, Affiliated Hospital of Guilin Medical University, Guilin, China; ^5^ Department of General Surgery, Hospital of Integrated TCM and Western Medicine, Southern Medical University, Guangzhou, China; ^6^ HBVtech, Germantown, MD, United States

**Keywords:** magnetic resonance imaging, hepatocellular carcinoma, nomogram, prediction, early recurrence, ablation technique

## Abstract

**Purpose:**

This study aimed to identify preoperative gadoxetic acid-enhanced MRI features and establish a nomogram for predicting early recurrence (≤ 2 years) of hepatocellular carcinoma (HCC) after ablation therapy.

**Methods:**

A total of 160 patients who underwent gadoxetic acid-enhanced MRI and ablation HCC therapy from January 2015 to June 2018, were included retrospectively and divided into a training cohort (n = 112) and a validation cohort (n = 48). Independent clinical risk factors and gadoxetic acid-enhanced MRI features associated with early recurrence were identified by univariate and multivariate logistic regression analysis and used for construction of a nomogram. The performance of the nomogram was evaluated by discrimination, calibration, and clinical utility.

**Results:**

Alpha-fetoprotein (AFP) level, tumor number, arterial peritumoral enhancement, satellite nodule and peritumoral hypointensity at hepatobiliary phases in the training cohort were identified as independent risk factors for early recurrence after ablation. A new nomogram that was constructed with these five features showed an area under the curve (AUC) of 0.843 (95%CI 0.771-0.916) and 0.835 (95%CI 0.713-0.956) in the training and validation cohort, respectively. The calibration curve and decision curve analysis (DCA) suggested that the nomogram had good consistency and clinical utility.

**Conclusions:**

A new nomogram that was constructed using four preoperative gadoxetic acid-enhanced MRI features and serum AFP level can predict the risk of early HCC recurrence after ablation therapy with AUC up to 0.843. The strong performance of this nomogram may help hepatologists to categorize patients’ recurrent risk to guide selecting treatment options and improve postoperative management.

## Introduction

Hepatocellular carcinoma (HCC) is the fourth most common cause for cancer-related death worldwide (1) and the second leading cause of years of life lost from cancers globally after lung cancer (2). Ablation therapy, together with resection and liver transplantation, constitutes three curative HCC treatments and has been increasingly applied for the HCC treatment because of minimally invasive nature. Several devices/agents can be employed for cancer ablation including radiofrequency ablation (RFA), microwave ablation (MWA), cryotherapy and percutaneous ethanol injection though RFA and MWA are mostly investigated in clinical practice. Previous studies showed that the 1-year recurrence rate of HCC after RFA or MWA varied between 10%-30% (3, 4). It is important for physicians to thoroughly analyze conditions of HCC patients and to rank likelihood for early HCC recurrence before procedure. As reported, the pathological, cellular molecular, and immunological classifications of HCC may help predict the therapeutic effectiveness and recurrence probability (5, 6). However, these analyses are invasive and may not be available before procedure. For instance, HCC histopathology is usually established postoperatively, some analyses require special testing platforms and are restricted to large medical centers. In addition, the current staging or scoring systems including the American Joint Committee on Cancer stage (AJCC) (7), Japan Integrated Staging (JIS) (8), Barcelona Clinic Liver Center stage (BCLC) (9) were initially designed to cover a full spectrum of HCC patients from early to advanced stages. For patients undergoing ablation therapy for very early or early stage of HCC, the above-mentioned scoring systems may offer a general assessment but lack fine resolution and clinical utility in predicting prognosis for those patients.

Gadoxetic acid (Gd-EOB-DTPA or Primovist) is a hepatocyte-specific contrast agent. Studies showed that hepatobiliary phases (HBP) of gadoxetic acid-enhanced MRI can significantly improve HCC diagnostic efficacy (10) by providing more detailed imaging profile of HCC lesion compared to other MR contrast agents or enhanced CT (11). The gadoxetic acid-enhanced MRI has been widely used for HCC diagnosis and monitoring pre and post procedures. High quality of imaging profile and ready availability from the gadoxetic acid-enhanced MRI make it an excellent candidate parameter for predicting the risk for postoperative HCC recurrence. Several studies reported that early HCC recurrence can be predicted by selected imaging features such as peritumoral enhancement in the arterial phase, satellite nodules, and non-smooth tumor margins from preoperative gadoxetic acid-enhanced MRI (12, 13). However, clinical values remain to be established because of significant variability and the lack of validation. In addition, most studies focused on the prediction after resection, not ablation, especially MWA.

Nomogram is a graphical display of regression model. It converts the complex mathematical regression functions into a scale composed of different factors that receive assigned scores. Occurrence probability of the endpoint event of interest can be quickly estimated by simply adding up the scores (14). The purpose of this study was to investigate whether the nomogram using preoperative gadoxetic acid-enhanced MRI can improve predicting HCC recurrence after ablation therapy.

## Materials and Methods

### Patients

**Figure 1:**
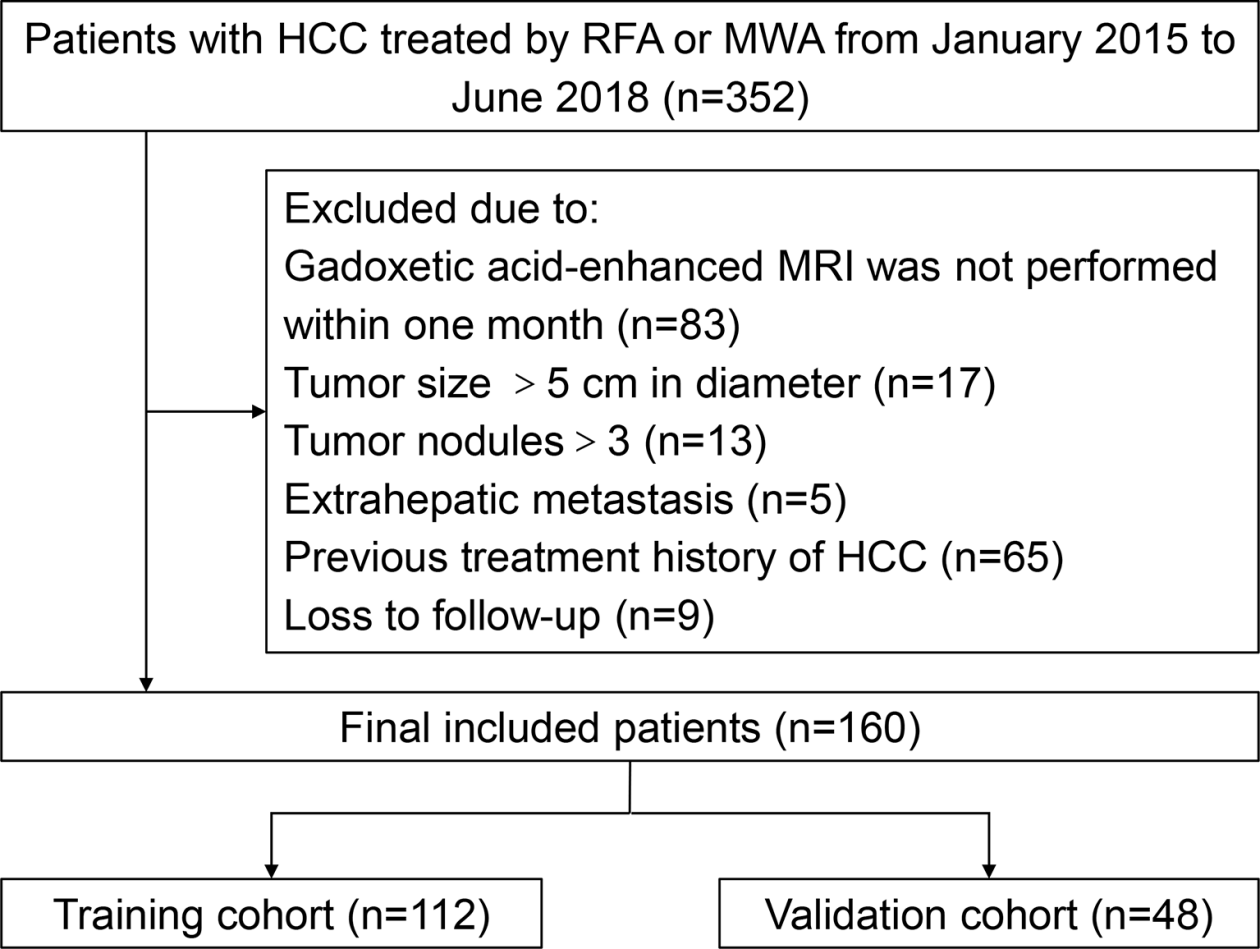
Flowchart of patient inclusion.

This study was approved by the Ethics Committee of Nanfang Hospital, Southern Medical University, Guangzhou, China. The informed consent was waived. A total of 352 HCC patients who were treated with ablation therapy during the period of January 2015 and June 2018 at Nanfang Hospital, were initially screened. Two inclusion criteria applied for enrollment: 1) cirrhotic patients who underwent preoperative gadoxetic acid-enhanced liver MRI were diagnosed with HCC; 2) HCC patients were treated with RFA or MWA. Patients with following characteristics were excluded: 1) without gadoxetic acid-enhanced MRI examination within one month before ablation therapy; 2) maximal diameter of tumor > 5cm; 3) tumor nodules > 3; 4) extrahepatic metastasis; 5) previous intervention therapy or hepatectomy; 6) loss to follow-up. There were 160 patients who were eligible for this study and divided into two cohorts according to the ablation therapy date at a ratio of 7:3. One was training cohort (n=112) to construct the nomogram model, and the other was validation cohort (n=48) to verify the performance of the model. The flowchart of patient selection was shown in **Figure 1**.

To evaluate the sample size for the study, the PASS software (15.0.5) was used to perform a power analysis based on the rate of early HCC recurrence of training group and validation group. The status of “arterial peritumoral enhancement”, “satellite nodule” and “peritumoral hypointensity on HBP” (variables in the nomogram model) of the subjects we included were used to compared. Normally, a power value greater than 0.8 suggests a sufficient sample size. The calculated power values were all over 0.95 in both groups for this study. Significant differences were computed with the two-sided Fisher’s Exact Test set at p <0.05. Thus, the sample sizes for the training and the validation groups were sufficient, meaning that the result and conclusion of this study were statistically significant.

### Ablation Therapy

RFA was carried out with a 200 W generator and a single electrode with a 17 gauge, 2-3 cm long exposed tip (RITA 1500X; Med Technology, Shenzhen, China). MWA was performed using a single water-cooled microwave system (KY-2000; Kangyou Medical, Nanjing, China), with a 10-100 W generator and a 15 gauge and 18 cm long cooled-shaft antennae. Real-time ultrasound was used to guide percutaneous ablation and multiple overlapping ablations were carried out. RFA was used when the tumor was adjacent to vital organs or high-risk structures, while MWA was used in cases of poor coagulation or large tumors. CEUS fusion imaging was carried out to immediately assess treatment response of ablation. The ablation procedure was considered successful when immediate post-ablation assessment showed that the ablation zone encompassed the visible mass with at least a 5 mm circumferential ablation margin. All patients underwent contrast-enhanced CT or MRI one month after ablation and the technical success of ablation therapy was defined with no observed enhancement at the arterial phase images at the ablation site (15), otherwise deemed incomplete ablation. For patients with incomplete ablation, repeated ablation was performed. If residual tumor remains after 2nd ablation treatment, the treatment was classified as a failure and the patient were recommended for other treatments and excluded from the study cohort.

### MRI Parameters and Process

All MR images were acquired using a 3.0 Tesla (T) whole-body MR imaging system (Intera Achieva 3T, Philips Healthcare, Best, the Netherlands) equipped with a dual source parallel radiofrequency transmission system and a quadrature body coil. For gadoxetic acid (Primovist; Bayer Healthcare, Berlin, Germany)–enhanced MR imaging, unenhanced, enhanced arterial phase (20–35 seconds), portal venous phase (60 seconds), delayed phase (3 minutes), and 20 minutes HBP images were obtained using a T1-weighted three-dimensional (3D) turbo-field-echo sequence (T1 high-resolution isotropic volume examination, THRIVE; Philips Healthcare). All patients were the cubital vein injected with gadoxetic acid at a total dose of 0.025 mmol/kg body weight into, followed by a 20-mL saline flush.

### Analysis of Gadoxetic Acid-Enhanced MRI

MRI analysis was performed by two radiologists (with more than ten years of experience in abdominal imaging) who were blinded to the clinical and laboratory information. The two reviewers independently evaluated whether the following imaging features were present: (1) tumor size: defined as the maximum diameter of a tumor; (2) tumor margin: smooth or non-smooth. ‘Non-smooth’ margin means irregular tumor margins with a tiny budding portion protruding into the liver parenchyma (16); (3) arterial rim enhancement: defined as the presence of irregular ring-like enhancement with relatively hypovascular central areas in the arterial phase (17); (4) arterial peritumoral enhancement: defined as enhancement outside the tumor margin with broad contact to the tumor border in the late arterial phase or early portal venous phase and becoming isointense with background liver parenchyma in the delayed phase (18); (5) satellite nodules: defined as nodules smaller than 2 cm with similar MR imaging characteristics and located within 2 cm from the main tumor borders (19); (6) tumor hypointensity on HBP: defined as the hypointensity area at HBP compared with the surrounding liver (20); (7) peritumoral hypointensity on HBP: defined as irregular hypointensity area of hepatic parenchyma located around the tumor on HBP (21). When disagreement occurred, a consensus was made through discussion with a third radiologist.

### Construction and Evaluation of Nomogram

Univariate and multivariate logistic regression analysis were performed to identify independent parameters, which were then employed to construct a nomogram. The discrimination performance of the nomogram was evaluated by Harrell’s c-index and receiver operator characteristic (ROC) curves (22). The predictive accuracy of the nomogram was validated by calibration curves and the Hosmer-Lemeshow test in the training and validation cohort (23). Per the sum of the nomogram prediction model scores, patients were divided into three groups (low-, medium- and high-risk groups), and the relapse-free survival (RFS) rate was calculated. Decision curve analysis (DCA) was performed to determine the clinical utility of the nomogram by quantifying the net benefits at different threshold probabilities (24).

### Follow-Up

Blood tests for serum alpha-fetoprotein (AFP) level and contrast-enhanced CT or gadoxetic acid-enhanced MR imaging were conducted during follow-up in the first month after ablation therapy, then every 3 months in year one and every 6 months thereafter (25). Early recurrence was defined as local tumor progression, intrahepatic distant recurrence, and extrahepatic metastasis within 2 years after ablation therapy. RFS was defined as the interval between ablation therapy and the date of tumor recurrence or the last follow-up date (without recurrence).

### Statistical Analysis

Statistical analyses were performed by SPSS (version 26, Chicago, IL, USA) and R software (version 3.6.2, http://www.Rproject.org). To compare baseline data between the training and the validation cohorts, the Student *t* test or Mann-Whitney *U* test for continuous variables, or the *χ*2 test or Fisher’s exact test for categorical variables, was selected. The cumulative RFS incidence was estimated using the Kaplan-Meier method. Univariate and multivariate logistic regression analyses were used to identify the independent risk factors for postoperative recurrence and multicollinearity test was performed. Cohen *κ* coefficient was used to evaluate the interobserver agreement on MR imaging features. The nomogram and calibration curves were plotted by using the “RMS” package. The ROC curves were plotted by using the “pROC” package and the DCA curves were plotted by using the “RMDA” package. For all tests, *P* < 0.05 was considered statistically significant.

## Results

### Baseline Characteristics Between the Training and Validation Cohorts

There were no significant differences in age, gender, origin of liver disease, Child-Pugh score, AFP level, ablation method, early recurrence, median recurrence time, tumor size and tumor number between the two cohorts, so were the MR imaging features including tumor margin, arterial rim enhancement, arterial peritumoral enhancement, satellite nodule, tumor hypointensity on HBP and peritumoral hypointensity at HBP. Among the included patients, the median follow-up was 108.4 weeks (**Table 1**).

**Table 1:** Demographic and clinical characteristics in training and validation cohort. (n=160).

Characteristics	Training cohort (n=112)	Validation cohort (n=48)	P value
Age	55.55±10.47	54.69±11.23	0.640
Sex			0.873
Men	99 (88.4%)	42 (87.5%)	
Women	13 (11.6%)	6 (12.5%)	
Origin of liver disease			0.056
HBV	101 (90.2%)	40 (83.3%)	
HCV	6 (5.4%)	1 (2.1%)	
Alcohol	2 (1.8%)	4 (8.3%)	
Others	3 (2.6%)	3 (6.3%)	
Child-pugh score			0.400
A	92 (82.1%)	42 (87.5%)	
B	20 (17.9%)	6 (12.5%)	
AFP (ng/ml)			0.414
<20	49 (43.8%)	19 (39.6%)	
20-400	45 (40.2%)	17 (35.4%)	
>400	18 (16.0%)	12 (25%)	
Ablation method			0.111
RFA	32 (28.6%)	8 (16.7)	
MWA	80 (71.4%)	40 (83.3)	
Early recurrence	63 (56.2%)	26 (54.2%)	0.808
Median duration for recurrence (weeks)	12 (IQR 7.0-26.0)	18 (IQR 8.75-44.75)	0.154
Tumor number			0.253
Solitary	69 (61.6%)	36 (75%)	
2 nodules	34 (30.4%)	12 (22.9%)	
3 nodules	9 (8.0%)	1 (2.1%)	
Tumor size (cm)	1.8 (IQR 1.3-2.58)	1.7 (IQR 1.2-2.6)	0.698
Tumor margin			0.260
Smooth	64 (57.1%)	32 (66.7%)	
Non-smooth	48 (42.9%)	16 (33.3%)	
Arterial rim enhancement			0.102
Absent	96 (85.7%)	36 (75%)	
Present	16 (14.3%)	12 (25%)	
Arterial peritumoral enhancement			0.259
Absent	79 (70.5%)	38 (79.2%)	
Present	33 (29.5%)	10 (20.8%)	
Satellite nodule			0.053
Absent	84 (75.0%)	43 (89.6%)	
Present	28 (25.0%)	5 (10.4%)	
Tumor hypointensity at HBP			0.317
Absent	4 (3.6%)	1 (2.1%)	
Present	108 (96.4%)	47 (97.9%)	
Peritumoral hypointensity on HBP			0.394
Absent	74 (66.1%)	35 (72.9%)	
Present	38 (33.9%)	13 (27.1%)	

HBV, hepatitis B virus; HCV, hepatitis C virus; AFP, Alpha-fetoprotein; RFA, radiofrequency ablation; MWA, microwave ablation; HBP, hepatobiliary phases.

### Characteristics of Local Ablation Therapy and Follow-Up

The location characteristics of HCC lesion in local ablation therapy are listed in **Supplementary Table 1**. In total, seven patients (4.38%) underwent 2nd ablation session to obtain technique efficacy according to the one-month follow-up procedure. One patient (0.63%) was excluded from our study due to the failure in the 2nd ablation session, then underwent resection was performed. No procedure-related death observed in this study. Of all the patients in this study, 89 (55.6%) experienced postoperative recurrence in two-year study, with a median 13-week duration for recurrence (IQR 7-31). The 1-year and 2-year RFS rates were 51.5% and 44.5%, respectively. In addition, types of HCC recurrence among the 159 patients who eventually achieved treatment success are list as the follows: 1) Local tumor progression: 12/159 (7.55%); 2) Intrahepatic distant recurrence: 72/159 (45.3%); and 3) Extrahepatic metastasis: 9/159 (5.67%).

### Identification of Independent Risk Factors for Early Recurrence After Ablation

**Figure 2:**
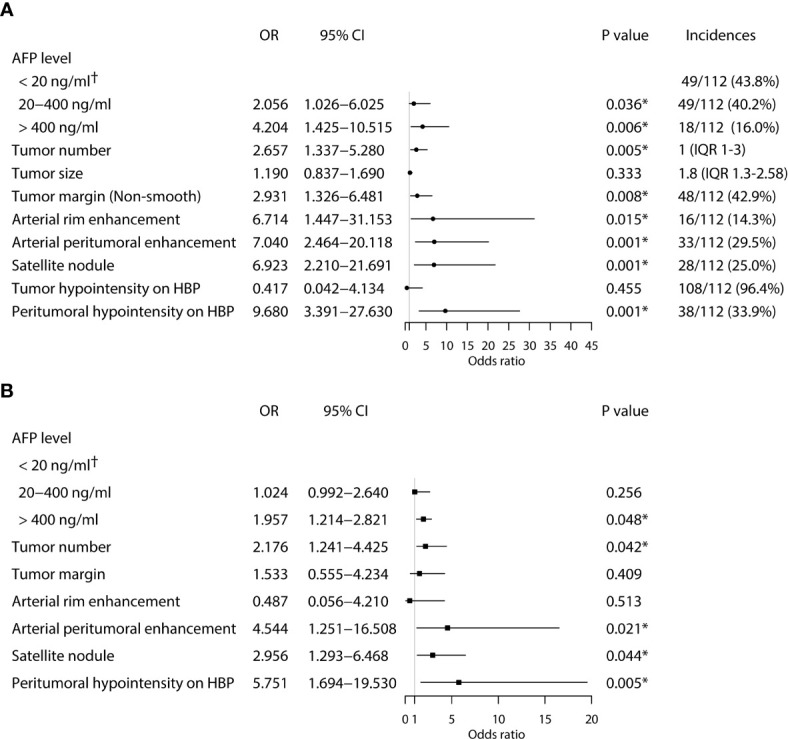
Univariate **(A)** and multivariate **(B)** analyses of independent risk factors associated with early HCC recurrence in the training cohort. (*Statistically significant results from logistic regression analysis; ^†^Used as the reference category).

**Figure 3:**
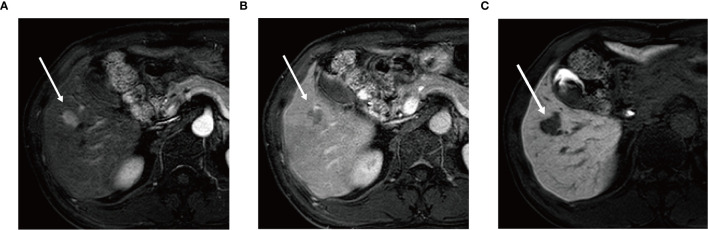
Gadoxetic acid-enhanced MR image **(A)** in arterial phase shows a 2.0 cm × 1.6 cm nodular lesion with arterial enhancement in hepatic segment V (arrow) and this nodule shows washout (arrow) in the portal venous phase **(B)**. In hepatobiliary phase **(C)** the lesion shows wedge-shaped peritumoral hypointensity area (arrow).

Univariate logistic regression analysis showed that AFP level, tumor number, tumor margin, arterial peritumoral enhancement, satellite nodule, and peritumoral hypointensity at HBP were associated with postoperative early recurrence in the training cohort. Then, these six parameters were included in the multivariate logistic regression analysis, identifying the following five markers as independent risk factors for early recurrence after ablation: AFP level (OR 1.957, 95%CI 1.214-2.821, *P* = 0.048), tumor number (OR 2.176, 95%CI 1.241-4.425, *P* = 0.042), arterial peritumoral enhancement (OR 4.544, 95%CI 1.251-16.508, *P* = 0.021), satellite nodule (OR 2.956, 95%CI 1.293-6.468, *P* = 0.044) and peritumoral hypointensity at HBP (OR 5.751, 95%CI 1.694-19.530, *P* = 0.005) (**Figure 2**). The multicollinearity test indicated no multicollinearity among the variables in the regression model, and the variance inflation factors (VIF) were 1.21, 1.30, 1.17, 1.36 and 1.20, respectively, which were all < 5. In addition, good consistency of three MR imaging features (*κ* = 0.61-0.8) was established among the observers (*κ* = 0.732 for arterial peritumoral enhancement, *κ* = 0.701 for satellite nodule, and *κ* = 0.755 for peritumoral hypointensity on HBP). Typical MR imaging features are illustrated in **Figure 3**.

### Development and Validation of the Nomogram

**Figure 4:**
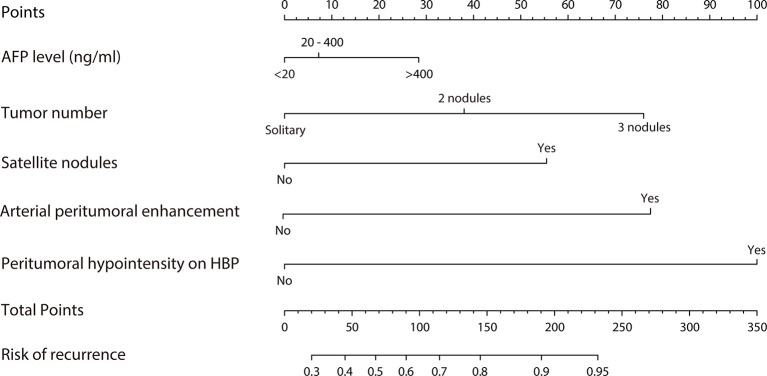
Nomogram predicting probability of early recurrence within 2 years after ablation therapy of HCC.

**Figure 5:**
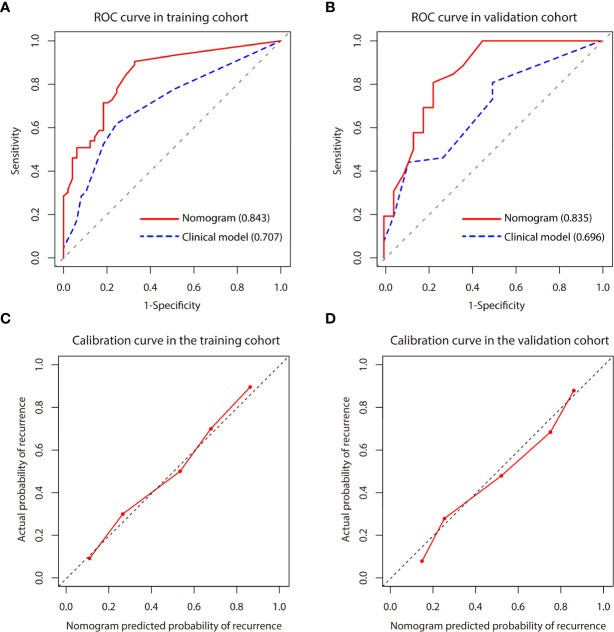
**(A, B)** Comparison of receiver operating characteristics (ROC) curves for predicting early recurrence between nomogram and clinical model in the training and validation cohort. The clinical model included AFP level and tumor number. **(C, D)** Calibration curve for the nomogram in the training and validation cohort. Calibration curves depict the agreement of the model between the predicted risks of early recurrence and the actual observed recurrence. X-axis represents the predicted probability of early recurrence. Y-axis represents the actual early recurrence, and the diagonal dashed line indicates the optimal prediction by a perfect model. The red solid line represents the performance of the nomogram, and the closer the red line is to the diagonal dashed line, the higher the prediction accuracy of the model.

A predictive nomogram model was established using the findings of multivariate logistic regression (**Figure 4**) and the detailed information is provided in **Supplementary Table 2**. Five variables employed by the nomogram included one clinical factors (AFP level) and four MR imaging features (tumor number, arterial peritumoral enhancement, satellite nodule and peritumoral hypointensity at HBP). The C-index was 0.808 and 0.745 (*P*>0.05) in the training and validation cohort, respectively. The nomogram yielded an AUC of 0.843 (95%CI 0.771-0.916) with 90.5% sensitivity of and 67.3% specificity for the training cohort, while displaying an AUC of 0.835 (95%CI 0.713-0.956) with 80.8% sensitivity of and 77.3% specificity (**Figures 5A, B**) for the validation cohort. However, if only two parameters of AFP level and tumor number employed (clinical model), the AUC values in the training cohort and the verification cohort were lowered to 0.707 and 0.696, respectively, which were worse off than the nomogram with gadoxetic acid-enhanced MRI features (*P* < 0.05) (**Figures 5A, B**). Comparisons of the predictive efficacy between the nomogram and the clinical model are included in Supplementary Material. Calibration curves (**Figures 5C, D**) and Hosmer-Lemeshow goodness of fit test indicated good consistency between the nomogram-predicted probability of recurrence after ablation and the actual rate of recurrence in both cohorts (*P* = 0.168 and *P* = 0.069).

### Evaluation of Risk Discrimination Ability by the Nomogram

**Figure 6:**
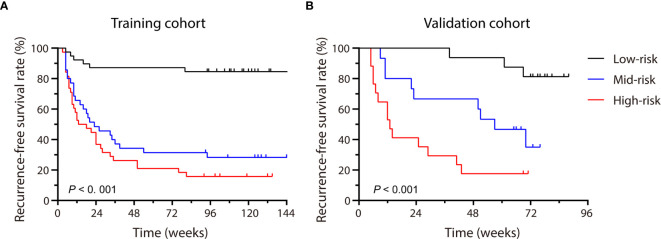
Patients were stratified by the scores calculated with the nomogram. The recurrence-free survival rate (RFS) of each group in the training cohort **(A)** and the validation cohort **(B)** were calculated, and significant differences in each risk group were observed by the Log-rank test (P < 0.001).

Each patient received a total score calculated through the nomogram, which ranged from 0-337 and 0-261 in the training and validation cohort, respectively. Patients were stratified into three groups (low-, medium- and high-risk groups) using 33.3% and 66.6% of the total score distribution range in the two cohorts (the cut-off scores were 7 and 138 for the training cohort and 13.6 and 100 for the validation cohort), and distinct RFS rate in each risk group was observed (*P* < 0.001, by log-rank test) (**Figures 6A, B**), demonstrating the risk discrimination ability by this nomogram.

### Clinical Application of the Nomogram

**Figure 7:**
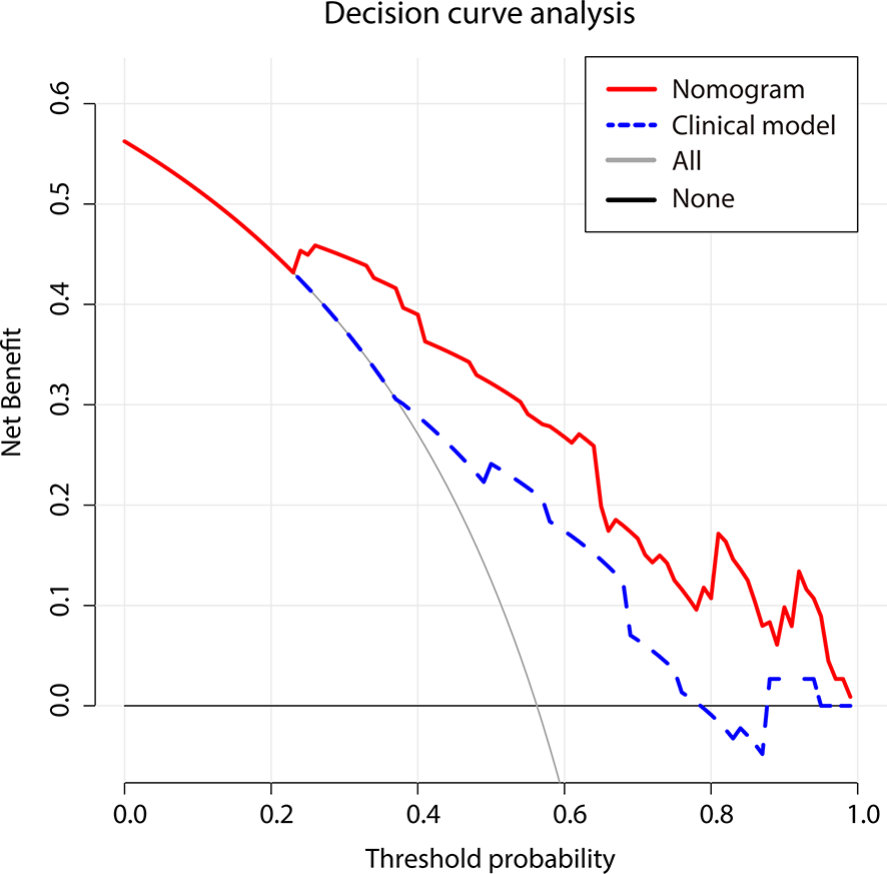
Decision curve analysis for two models. The use of the nomogram for early recurrence prediction provides more benefit than two extreme conditions [the treat-all-patients scheme (gray line) and the treat-none scheme (horizontal black line)]. Nomogram (red line) receives a higher net benefit than the model based on clinical factors (blue line) across a full range of reasonable threshold probabilities.

DCA curve (**Figure 7**) showed that the nomogram’s net benefit was higher than both extreme curves when the threshold probability set between 24% and 99%, and higher than the two-factor based model, suggesting potential clinical benefits provided by this nomogram.

## Discussion

This study found that arterial peritumoral enhancement, satellite nodule, and peritumoral hypointensity at HBP detected by gadoxetic acid-enhanced MRI were independent risk factors for early recurrence after HCC ablation therapy. We established a nomogram prediction model by combining these three risk factors with AFP level and tumor number. This model achieved a remarkable AUC of 0.843 as applied for this study population. To our knowledge, this is the first time that a nomogram using preoperative gadoxetic acid-enhanced MRI features and serum AFP level was constructed for predicting early HCC recurrence after RFA or MWA. If confirmed in future studies, this model represents a new progress in improving current prediction systems for early HCC recurrence after ablation.

In recent years, many studies have evaluated different models for predicting the risk of postoperative HCC recurrence using various clinical and radiologic parameters, reporting diverse performances. For instance, An C et al. (26) established a nomogram with incorporated tumor size, tumor number, Child-Pugh score, platelet count and alanine aminotransferase to predict the risk of local tumor progression in HCC after MWA, showing C-index of 0.799 and 0.732 in the training and validation datasets, respectively. However, the study did not include radiologic features of HCC, which may indicate aggressiveness, invasiveness, and stage of HCC that may exert significant impact on prognosis. For this reason, our nomogram model gave overwhelming weight on gadoxetic acid-enhanced MRI features. As expected, this new model fared very well in risk prediction in our two cohorts, and C-index was increased to 0.808 (with gadoxetic acid-enhanced MRI features) from 0.694 (without them) in the training cohort and increased to 0.745 from 0.679 in the verification cohort. Another study reported the performance of a nomogram that included clinical factors and CT-based radiological features in predicting the risk of solitary HCC recurrence (27), with an AUC of 0.639 (95% CI: 0.577–0.701). Although its diagnostic performance was higher than the traditional staging system (C-index: 0.552 for TNM and 0.547 for BCLC), the overall performance remained to be improved. Our model did improve it with an AUC of 0.843, suggesting that gadoxetic acid enhanced MRI contrast imaging that detects tumor boundaries indicating invasiveness is of greater prediction values compared CT imaging (13). Moreover, the AUC value from the validation cohort provided a first verification of the performance.

This new nomogram included serum AFP level, tumor number, and three gadoxetic acid-enhanced MRI features (arterial peritumoral enhancement, satellite nodule and peritumoral hypointensity at HBP). Serum AFP level and tumor number represent two consensus biomarkers associated with HCC recurrence. However, unlike previous studies (28–30), tumor size was not an independent risk factor for HCC recurrence in this study, which probably reflected that this study employed the strict inclusion criteria and ablation therapy was performed for very early and early stage of HCC. In fact, the median tumor size in this study was 1.8 cm (IQR 1.3-2.6 cm). Our findings that arterial peritumoral enhancement, satellite nodule and peritumoral hypointensity at HBP were associated with postoperative HCC recurrence was consistent with other studies (13, 31). These three features indicate potential microvascular invasion (MVI), a higher risk of tumor recurrence (18, 32). Moreover, a recent multi-center study compared surgical resection and RFA in patients with HCC based on the risk of MVI, showing that AFP, PIVKA-II, arterial peritumoral enhancement, and hepatobiliary peritumoral hypointensity on MRI were associated with MVI, which was in line with our results (33). In this study, tumor margin and arterial rim enhancement were not an independent risk factor either (OR 1.533, *P* = 0.409). We note that published studies offered opposite views on the relevance of these two markers to HCC recurrence (13, 28, 32).

Most published studies tried to predict the risk for resected HCC recurrence. This study modeled the risk for RFA or MWA treated HCC patients. Increasing evidence suggests no significant difference in efficacy between ablation and resection for very early and early stage of HCC (34, 35). The EASL guidelines recommends RFA and MWA as first-line treatment for BCLC 0 and stage A patients (36), and we expect an expanding ablation application in HCC treatment. Though a small number of studies evaluated the performance using preoperative MRI features in predicting the risk for HCC recurrence after ablation, all studies used the RFA treated patients (37, 38). This study fills void with regard to the recurrent risk for MWA treated patients.

In addition, compared with the radiomics based on machine learning, the imaging features included in this study can be easily interpreted and utilized by clinicians, and the prediction appeared to be reproducible. Decision curve analysis suggests a greatly potential clinical application value by this nomogram. For instance, preoperative HCC patients can be categorized into different risk groups based on the score assigned by this nomogram, providing an early opportunity to identify patients with higher risk for recurrence and to develop individualized therapies to reduce tumor recurrence and improve outcome. For example, when a patient’s nomogram score reached > 150 points, this patient may face high risk (>80% chance) for HCC recurrence within 2 years after ablation. A recommendation of liver transplantation or ablation combined with TACE or adjuvant chemotherapy, other than a simple ablation should be made.

This study carried a few limitations. First, this was a single-center study with HCC patient population dominantly associated with chronic HBV infection. Our findings require further validation in diverse HCC patient populations that undergo ablation therapy, as well as in MR scanners other than Philips. Second, although specific tumor location such as periportal or subphrenic location is known to be associated with a higher risk of local tumor progression, these factors were not taken into consideration in this study. Third, another important tumor marker, PIVKA-II, which is known to be a risk factor for tumor recurrence and is mentioned in guidelines including the APASL and EASL (36, 39). Unfortunately we were unable to include it as not widely performed in our hospital. A further study is warranted for a comprehensive understanding of the potential factors affecting early HCC recurrence.

## Conclusions

In conclusion, a new nomogram that incorporated unique preoperative gadoxetic acid-enhanced MRI features was developed and remarkably improved predicting the risk of early HCC recurrence after ablation therapy compared to the model without gadoxetic acid-enhanced MRI features. This novel model, if further validated in large and diverse HCC patient populations, may enable physicians to relatively accurately estimate potential risk for early HCC recurrence and guide selecting treatment options and personalize postoperative management plan to improve clinical outcomes of HCC treatment.

## Data Availability Statement

The original contributions presented in the study are included in the article/**Supplementary Material**. Further inquiries can be directed to the corresponding authors.

## Ethics Statement

The studies involving human participants were reviewed and approved by the Ethics Committee of Nanfang Hospital, Southern Medical University, Guangzhou, China. Written informed consent for participation was not required for this study in accordance with the national legislation and the institutional requirements.

## Author Contributions

Study design: YPZ, YYZ, YKX. Data collection: YDS, LD, CRT, ML. Data analysis: CGH, YDS, JZ. Writing: CGH, YDS. Revising: YPZ, YYZ, WJL. All authors contributed to the article and approved the submitted version.

## Funding

This study was supported by the grant from the National Natural Science Foundation of China (81772923). The funding agencies has no role in study design, data collection and analysis, decision to publish, or preparation of the manuscript.

## Conflict of Interest

Y-YZ is the founder and CSO of HBVtech.

The remaining authors declare that the research was conducted in the absence of any commercial or financial relationships that could be construed as a potential conflict of interest.
