# Supplementary material for: Preoperative Gadoxetic Acid-Enhanced MRI Based Nomogram Improves Prediction of Early HCC Recurrence After Ablation Therapy
Source: Front Oncol. 2021 May 21;11:649682. doi: 10.3389/fonc.2021.649682 (PMC8176857; doi:10.3389/fonc.2021.649682)
Supplement: Supplementary file 1 [file DataSheet_1.docx]

Supplementary Table 1. Location characteristics of HCC lesion in this study (n=160).

| Segmental location | Proportion |
| --- | --- |
| Ⅰ | 2 (1.25%) |
| Ⅱ | 19 (11.88%) |
| Ⅲ | 20 (12.50%) |
| Ⅳ | 26 (16.25%) |
| Ⅴ | 12 (7.50%) |
| Ⅵ | 18 (11.25%) |
| Ⅶ | 23 (14.38%) |
| Ⅷ | 40 (25.00%) |
| Left hepatic lobe | 67 (41.88%) |
| Right hepatic lobe | 93 (58.13%) |

Supplementary Table 2. Logistic regression model in the training cohort

|  | Coefficient |
| --- | --- |
| Intercept | -1.4857 |
| AFP level (20-400 ng/ml) | 0.2001 |
| AFP level (> 400 ng/ml)  Tumor number  2 nodules  3 nodules | 0.7029  0.8620  1.4529 |
| Satellite nodules | 0.7492 |
| Arterial peritumoural enhancement | 1.5158 |
| Peritumoural hypointensity at HBP | 1.7765 |

Supplementary Table 3. Predictive efficacy of the nomogram and the model without gadoxetic acid-enhanced MRI features

| Models | Training cohort | | | Validation cohort | | |
| --- | --- | --- | --- | --- | --- | --- |
|  | AUC(95%CI) | Sensitivity% | Specificity% | AUC(95%CI) | Sensitivity% | Specificity% |
| Nomogram | 0.843(0.771-0.916) | 90.5 | 67.3 | 0.835 (0.713-0.956) | 80.8 | 77.3 |
| Clinical factors | 0.707(0.610-0.804) | 61.9 | 75.5 | 0.696(0.546-0.846) | 80.8 | 50.0 |
